# ﻿A preliminary molecular phylogeny of the family Hydroptilidae (Trichoptera): an exploration of combined targeted enrichment data and legacy sequence data

**DOI:** 10.3897/zookeys.1111.85361

**Published:** 2022-07-11

**Authors:** Robin E. Thomson, Paul B. Frandsen, Ralph W. Holzenthal

**Affiliations:** 1 University of Minnesota, Department of Entomology, 1980 Folwell Ave., St. Paul, MN 55108, USA University of Minnesota St. Paul United States of America; 2 Brigham Young University, Department of Plant and Wildlife Sciences, Provo, UT 84602, USA Brigham Young University Provo, UT United States of America

**Keywords:** Caddisfly, diversity, molecular dataset, systematics

## Abstract

Hydroptilidae is an extremely diverse family within Trichoptera, containing over 2,600 known species, that displays a wide array of ecological, morphological, and habitat diversity. However, exploration into the evolutionary history of microcaddisflies based on current phylogenetic methods is mostly lacking. The purpose of this study is to provide a proof-of-concept that the use of molecular data, particularly targeted enrichment data, and statistically supported methods of analysis can result in the construction of a stable phylogenetic framework for the microcaddisflies. Here, a preliminary exploration of the hydroptilid phylogeny is presented using a combination of targeted enrichment data for ca. 300 nuclear protein-coding genes and legacy (Sanger-based) sequence data for the mitochondrial COI gene and partial sequence from the 28S rRNA gene.

## ﻿Introduction

Caddisflies, or Trichoptera, are a diverse order of insects with more than 16,000 described species and 100s of new species awaiting placement and description (Morse 1999; Holzenthal 2009; Holzenthal et al. 2015). Moth-like as adults, Trichoptera are closely related to Lepidoptera (butterflies and moths). Larvae are aquatic and produce silk, which is used to construct a wide variety of portable cases and filtering nets (Wiggins 1996, 2004).

As the common name “microcaddisfly” suggests, Hydroptilidae represent the smallest family in the order in terms of body size, with adults ranging from between 1.5 mm to usually no more than 5 mm in length (Holzenthal et al. 2007b). Microcaddisflies are extremely diverse; larvae occur in a wide array of aquatic habitats, display numerous feeding patterns, and last instars construct a variety of larval cases known collectively for the family as “purse-cases” and exhibit an interesting hypermetamorphosis observed within Trichoptera only in Hydroptilidae and its sister group, Ptilocolepidae (Nielsen 1948; Wells 2010).

In terms of species diversity, Hydroptilidae is the largest family in the order Trichoptera, including more than 2,600 species in 76 genera (including three fossil genera) and six subfamilies, found in all faunal regions of the world (Marshall 1979; Morse 1999; Holzenthal et al. 2011) (Table 1). Of the six subfamilies, two are largely endemic to the Neotropical faunal region (Leucotrichiinae and Neotrichiinae), though some of the included species are distributed well into North America. Ochrotrichiinae is distributed primarily in the Neotropics, with two genera occurring in Australasia. Hydroptilinae occurs in the Old World, but also includes two large cosmopolitan genera (*Hydroptila* and *Oxyethira*) and several genera endemic to the Australasian or Afrotropical faunal regions. The subfamily Orthotrichiinae is small, but includes the cosmopolitan genus *Orthotrichia*, while the subfamily Stactobiinae is a varied collection of genera that are either endemic to a particular region or occur in a wider distribution throughout multiple regions. The closely related Ptilocolepidae are a small family, formerly considered to be a subfamily within Hydroptilidae, which currently contains the genera *Ptilocolepus* and *Palaeagapetus* distributed throughout the Holarctic faunal region. Since being elevated to family status (Malicky 2001), the placement of Ptilocolepidae and its relationship with Hydroptilidae has been contentious (Holzenthal et al. 2007a; Malicky 2008; Thomas et al. 2020).

**Table 1.** Currently recognized genera of Hydroptilidae and Ptilocolepidae and family-group classification.

Family	Subfamily	Tribe	Genera	
Hydroptilidae	Hydroptilinae	–	*Acanthotrichia*	*Microptila*
			*Acritoptila*	*Missitrichia*
			*Aenigmatrichia*	*Mulgravia*
			*Agraylea*	*Oxyethira*
			*Allotrichia*	*Paroxyethira*
			*Austratrichia*	*Paucicalcaria*
			*Cyclopsiella*	*Sutheptila*
			*Dhatrichia*	*Tangatrichia*
			*Hellyethira*	*Tricholeiochiton*
			*Hydroptila*	*Ugandatrichia*
			*Jabitrichia*	*Vietrichia*
			*Kholaptila*	*Wlitrichia*
			*Maeyaptila*	*Xuthotrichia*
	Leucotrichiinae	Alisotrichiini	*Alisotrichia*	*Cerasmatrichia*
			*Byrsopteryx*	*Mejicanotrichia*
			*Celaenotrichia*	*Scelobotrichia*
		Leucotrichiini	*Acostatrichia*	*Costatrichia*
			*Anchitrichia*	*Leucotrichia*
			*Ascotrichia*	*Peltopsyche*
			*Betrichia*	*Tupiniquintrichia*
			*Ceratotrichia*	*Zumatrichia*
	Neotrichiinae	–	*Kumanskiella*	*Neotrichia*
			*Mayatrichia*	*Taraxitrichia*
	Ochrotrichiinae	–	*Angrisanoia*	*Nothotrichia*
			*Caledonotrichia*	*Ochrotrichia*
			*Dibusa*	*Ragitrichia*
			*Maydenoptila*	*Rhyacopsyche*
			*Metrichia*	
	Orthotrichiinae	–	*Ithytrichia*	*Saranganotrichia*
			*Orthotrichia*	
	Stactobiinae	–	*Bredinia*	*Pseudoxyethira*
			*Catoxyethira*	*Orinocotrichia*
			*Chrysotrichia*	*Plethus*
			*Flintiella*	*Stactobia*
			*Maetalaiptila*	*Stactobiella*
			*Niuginitrichia*	*Tizatetrichia*
Hydroptilidae, incertae sedis	–	–	*Burminoptila* ♰	*Macrostactobia*
			*Dicaminus*	*Novajerseya* ♰
			*Electrotrichia* ♰	*Orphninotrichia*
Ptilocolepidae	–	–	*Palaeagapetus*	*Ptilocolepus*

**Figure 1.**
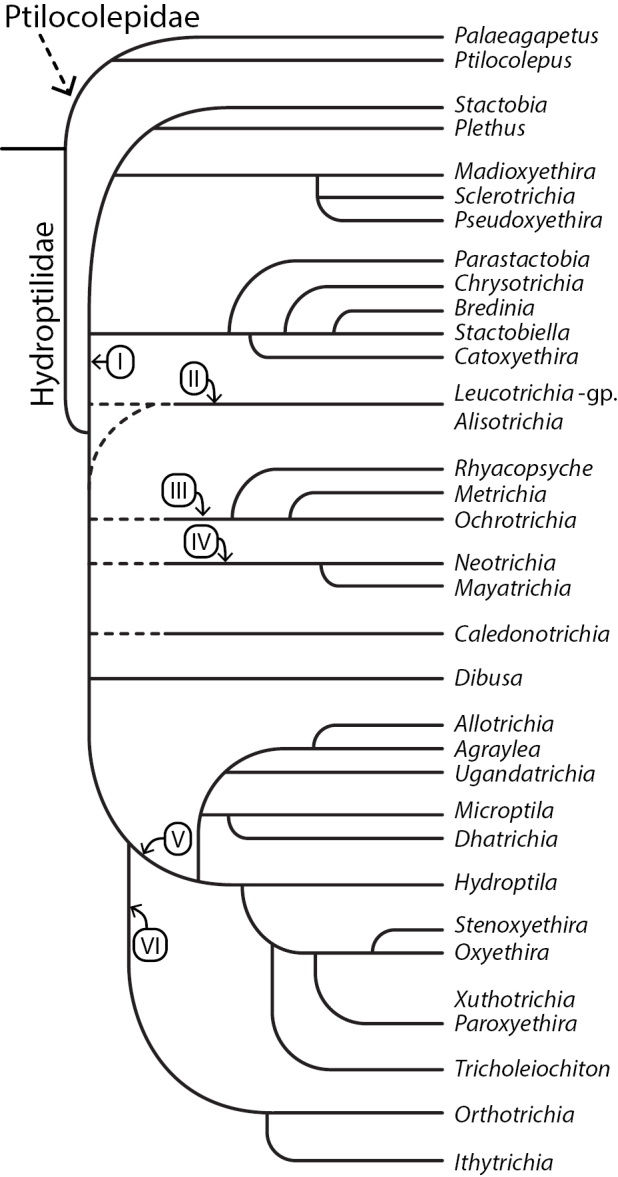
Phylogeny of Hydroptilidae, re-drawn from Marshall (1979). Based on morphological data; generic relationships (**I**Stactobiini**II**Leucotrichiini**III**Ochrotrichiini**IV**Neotrichiini**V**Hydroptilini**VI**Orthotrichiini).

Marshall (1979) provided the first comprehensive review of Hydroptilidae at the generic level, including the 42 genera known at the time. The morphology-based phylogeny she proposed was not based on any statistical analyses and therefore offered no support values for any of the proposed relationships (Fig. 1). The only other attempt to provide a family-wide systematic framework for Hydroptilidae was that of Oláh and Johanson (2011), a work in which they described many new species and updated the genera to be included in each subfamily. Several tables were provided, containing either features or character states of species groups, subgenera, or generic clusters; there was no discussion presented regarding the information outlined in the tables. As interpreted from the tables, several genera were transferred between subfamilies or moved from incertae sedis status, but no phylogeny or hypotheses of relationships were included.

Several subfamilies have a history of being difficult to unite by any morphological features. For example, various Trichoptera researchers have made published comments regarding the difficulty in uniting the subfamily Stactobiinae or finding any derived characters exclusive to the group (Wells 1990; Bowles et al. 1999; Malicky and Chantaramongkol 2007). Leucotrichiinae is the only subfamily that has undergone a detailed phylogenetic analysis; a relatively recent assessment confirmed the monophyly of the family and generic assignment to two newly established tribes (Leucotrichiini and Alisotrichiini) for the first time (Santos et al. 2016).

A stable framework based on statistically-supported phylogenetic methods is needed to consistently define taxa and provide context for how they relate to each other and are arranged within the family overall. Wiggins (2004) suggested that a thorough analysis of phylogenetic relationships is important for taxonomic and systematic progression; Hydroptilidae has consistently been supported as monophyletic in studies of family relationships across Trichoptera, but analysis of the relationships within this hyperdiverse family has long been neglected. The microcaddisflies have shown a long history of instability and tenuous placement within Trichoptera but tended to occur with several families in various arrangements near the base of Trichoptera (Ross 1967; Weaver 1984; Weaver and Morse 1986; Wiggins and Wichard 1989; Frania and Wiggins 1997; Ivanov 1997; Kjer et al. 2001; Malm et al. 2013). In the most recent study using molecular data to explore the relationships among the caddisfly families, Hydroptilidae were grouped with the suborder Integripalpia in an arrangement that was supported by different methods of analysis and independent datasets (Thomas et al. 2020). A stable phylogeny for Hydroptilidae would also be useful for larger questions applied to the order Trichoptera. Targeted enrichment has emerged as a useful and popular tool for sequencing many genes from museum specimens. It allows for sequencing across many hundreds of genes, even for specimens with degraded DNA (Lemmon et al. 2012). Recently, Deng and colleagues applied this approach to the trichopteran genus *Himalopsyche* (Deng et al. 2021). This preliminary study acts as a proof-of-concept that targeted enrichment sequence data using the previously published Trichoptera probe set can be successfully obtained from hydroptilid specimens and, when used in tree construction, can successfully recover expected clades, and produce a phylogeny with high support values. Our specific objectives are to provide a preliminary analysis of the monophyly of Hydroptilidae, Ptilocolepidae, and the hydroptilid subfamilies in their current classification system, and to explore the relationships within and between these taxa.

## ﻿Materials and methods

### ﻿Selection of taxa

The taxa included in this study were chosen to represent the overall taxonomic diversity of the family Hydroptilidae by including examples of all subfamilies and as many genera as possible. A list of the specimens from which DNA was sequenced for this study is presented in Table 2.

**Table 2.** Determination, depository, and sequencing method of specimens included in phylogenetic analyses. “Composite” refers to instances in which we combined sequence data for two closely related species in the same genus for the sake of matrix completeness.

		Depository	Targeted Enrichment	Sanger	Composite
**INGROUP**					
**Hydroptilidae**					
**Hydroptilinae**					
*Agraylea*	cognatella	ZMUB		X	
	multipunctata	RUIC	X	X	
	sexmaculata	RUIC		X	
	*saltesea*	RUIC		X	
	cf.saltesea	BOLD		X	
*Allotrichia*	vilnensis	BOLD	X	X	
*Hellyethira*	simplex	UMSP		X	
*Hydroptila*	ajax	BOLD		X	
	albicornis	BOLD		X	
	ampoda	BOLD		X	
	argosa	BOLD		X	
	consimilis	BOLD		X	
	coweetensis	BOLD		X	
	delineata	BOLD		X	
	forcipata	ZMUB		X	
	gunda	CUAC		X	
	hamata	CUAC		X	
	jackmanni	BOLD		X	
	losida	UMSP		X	
	oguranis	UMSP		X	
	rono	BOLD		X	
	scamandra	UMSP		X	X
	tineoides	ZMUB	X	X	
	vectis	RUIC		X	X
	xera	BOLD		X	
*Oxyethira*	absona	RUIC		X	
	bidentata	RUIC		X	
	frici	ZMUB		X	
	grisea	CUAC		X	
	janella	CUAC		X	
	rivicola	RUIC		X	
	rossi	RUIC		X	
*Paroxyethira*	hendersoni	NMNH		X	
	tillyardi	NMNH		X	
*Ugandatrichia*	maliwan	RUIC		X	
	sp.	RUIC		X	
**Leucotrichiinae**					
*Abtrichia*	antennata	UMSP		X	
	squamosa	UMSP		X	
	veva	NMNH		X	
*Alisotrichia*	fundorai	NMNH		X	
	*hirudopsis aitija*	NMNH		X	
*Anchitrichia*	duplifurcata	UMSP		X	
	spangleri	RUIC		X	
*Ascotrichia*	surinamensis	NMNH		X	X
	sp.	RUIC		X	X
*Byrsopteryx*	abrelata	UMSP		X	
	chaconi	UMSP		X	
	esparta	UMSP		X	
	gomezi	UMSP	X	X	
	solisi	UMSP		X	
	tapanti	UMSP		X	
	tica	UMSP		X	
*Celaenotrichia*	edwardsi	BOLD		X	
*Cerasmatrichia*	spinosa	BOLD	X	X	
	trinitatis	NMNH		X	
*Ceratotrichia*	flavicoma	NMNH		X	
*Leucotrichia*	fairchildi	RUIC		X	X
	pictipes	RUIC		X	X
	sarita	NMNH	X	X	
*Zumatrichia*	anomaloptera	NMNH		X	
	diamphidia	RUIC		X	X
	rhamphoides	UMSP		X	X
**Neotrichiinae**					
*Mayatrichia*	ayama	NMNH		X	
	rualda	UMSP		X	
*Neotrichia*	feolai	BOLD	X	X	
	minutisimella	UMSP		X	
	vibrans	UMSP		X	
**Ochrotrichiinae**					
*Dibusa*	angata	NMNH		X	
*Metrichia*	fontismoreaui	NMNH		X	
	neotropicalis	UMSP		X	
	nigritta	UMSP		X	
	patagonica	UMSP		X	
	platigona	NMNH		X	
	spica	UMSP		X	
	yalla	NMNH		X	
*Nothotrichia*	cautinensis	BOLD		X	
*Ochrotrichia*	alsea	UMSP		X	
	dactylophora	BOLD		X	
	eliaga	RUIC		X	
	logana	RUIC		X	
	limonensis	UMSP		X	
	oregona	UMSP		X	
	panamensis	RUIC		X	
	tarsalis	UMSP		X	
	tenanga	UMSP		X	
*Rhyacopsyche*	andina	UMSP		X	
	dikrosa	UMSP		X	
	hagenii	UMSP		X	
	mexicana	UMSP		X	
**Orthotrichiinae**					
*Ithytrichia*	lamellaris	USDC		X	
*Orthotrichia*	curvata	BOLD	X	X	
	tragetti	BOLD	X	X	
**Stactobiinae**					
*Stactobia*	makartshenkoi	NMNH		X	
	nybomi	NMNH		X	
*Stactobiella*	delira	UMSP	X	X	
	*martynovi*	RUIC		X	
	palmata	BOLD		X	
	tshistjakovi	UMSP		X	
**Incertae sedis**					
*Orphninotrichia*	squamosa	UMSP		X	
**Ptilocolepidae**					
*Palaeagapetus*	celsus	RUIC		X	
	nearcticus	BOLD		X	
	ovatus	NMNH		X	
*Ptilocolepus*	extensus	USDC	X	X	
	granulatus	RUIC		X	
**OUTGROUP**					
**Glossosomatidae**					
*Agapetus*	pinatus	RUIC		X	
*Agapetus*	tomus	BOLD	X	X	
*Anagapetus*	bernea	BOLD		X	
	debilis	RUIC		X	
*Cariboptila*	aurulenta	BOLD		X	
*Culoptila*	hamata	RUIC		X	
*Glossosoma*	nigrior	RUIC		X	
*Padunia*	jeanae	RUIC		X	
*Protoptila*	laterospina	BOLD		X	
	tenebrosa	RUIC		X	
**Hydrobiosidae**					
*Apatanodes*	sociatus	BOLD		X	
*Apsilochorema*	gisbum	RUIC		X	
*Atopsyche*	callosa	RUIC		X	
	sp.	RUIC		X	
*Taschorema*	evansi	RUIC		X	
*Ulmerochorema*	onychion	RUIC		X	
	rubiconum	BOLD		X	
**Rhyacophilidae**					
*HImalopsyche*	malenada	BOLD		X	
*Rhyacophila*	brunnea	RUIC	X	X	
	coloradensis	RUIC	X	X	
	fuscula	RUIC		X	
**Phryganeidae**					
*Yphria*	californica	BOLD	X	X	
**Leptoceridae**					
*Leptocerus*	americanus	BOLD	X	X	
**Sericostomatidae**					
*Myotrichia*	murina	BOLD		X	
**Limnephilidae**					
*Limnephilus*	externus	BOLD		X	

### ﻿Targeted enrichment taxon sampling

#### Ingroup

We sequenced eleven ingroup species of microcaddisflies using targeted enrichment sequencing (Lemmon et al. 2012), including ten species from the family Hydroptilidae and one species from the family Ptilocolepidae. These taxa represent five of six subfamilies, with the exception of Ochrotrichiinae.

#### Outgroup

We selected an additional five species from four different families as outgroups, including representatives from Rhyacophilidae, Glossosomatidae, Phryganeidae, and Leptoceridae.

### ﻿Sanger sequencing taxon sampling

#### Ingroup

The ingroup, Hydroptilidae and Ptilocolepidae, included 104 species units representing a total of 32 genera. Representatives from both ptilcolepid genera and all six traditionally recognized hydroptilid subfamilies were included as ingroup taxa. As many genera from each subfamily were obtained as possible and all taxa from which DNA was successfully sequenced and amplified were included in the dataset. Large subfamilies and genera, such as Hydroptilinae, *Hydroptila*, and *Oxyethira*, were sampled more rigorously to account for high species richness. There were some taxa included in the targeted enrichment taxon sampling for which no Sanger sequencing data existed. For the fastRFS analysis, we assigned those taxa to the closest available taxon with available Sanger sequencing data based on their classification (Table 2).

#### Outgroup

The outgroup consisted of 25 species including members from the families Glossosomatidae, Hydrobiosidae, Rhyacophilidae, Phryganeidae, Leptoceridae, Sericostomatidae, and Limnephilidae.

### ﻿Depositories

Specimens sequenced for this study were obtained from the National Museum of Natural History, Washington, DC, USA (**NMNH**); University of Minnesota Insect Collection, St. Paul, MN, USA (**UMSP**), Clemson University Arthropod Collection, Clemson, SC, USA (**CUAC**); Zoological Museum, University of Bergen, Bergen, Norway (**ZMUB**); Rutgers University Entomology Museum, New Brunswick, NJ, USA (**RUIC**); and Departmento de Zoología y Antropología Física, Universidad de Santiago de Compostela, Santiago de Compostela, Spain (**USDC**). Additionally, Dave Ruiter, Grants Pass, Oregon, USA; Alice Wells, Australian Biological Resources Study, Canberra, ACT, Australia; and Tomiko Ito, Hokkaido Aquatic Biology, Hokkaido, Japan generously donated several specimens from their private collections to UMSP. Voucher materials from specimens that were successfully sequenced are deposited at the NMNH, UMSP, CUAC, ZMUB, and USDC. All specimens from which DNA was sequenced for this study were affixed with a barcode label (4 mil polyester, 8 × 14 mm, code 49) bearing a unique alphanumeric sequence beginning with the prefix UMSP. The prefix does not imply ownership by UMSP, but only indicates that the specimen was databased at that collection and to provide unique identification code (UID) for entry into a database. Specimen-level taxonomic, locality, and other information are stored in the University of Minnesota Insect Collection database using the software Specify 6.7.02 (Specify Collections Consortium 2022).

### ﻿DNA Sequences

To create a scaffold of phylogenetic relationships among subfamilies, we used targeted enrichment to capture 302 genes across a subset of the taxa sampled (Table 2).

### ﻿DNA extraction

DNA was extracted from pinned or 95% ethanol-preserved museum specimens. In cases of ethanol-preserved specimens, attempts were made to use the most recently collected specimens available. Due to the physically minute size of individual specimens, the head, thorax, and legs were all taken for extraction. In all cases, male genitalia were retained as specimen voucher material, and the specimen data were entered into the UMSP Specify database. Genitalia were prepared for preservation following the lactic acid method, procedures for which are explained in detail by Blahnik et al. (2007). DNA was extracted in either the laboratory of Dr. Karl Kjer, Rutgers University, or of Dr. Susan Weller, University of Minnesota. DNA extraction was completed using the DNEasy Blood and Tissue Kit (Qiagen, Inc.) with 20 μl of Proteinase K (Qiagen, Inc.).

### ﻿Targeted enrichment

We used the Trichoptera probe set published in Deng et al. (2021) for the targeted enrichment analyses. Following DNA extraction, quantification, targeted enrichment, library preparation, and DNA sequencing were conducted off-site by Rapid Genomics. For sequencing, paired-end 2 × 150 bp reads were sequenced on an Illumina NovaSeq instrument.

### ﻿PCR and Sanger sequencing

Targeted gene sequences for COI and partial 28S were amplified using polymerase chain reaction (PCR) with Accuzyme Mix (Bioline) and the primers listed in Table 3. An additional 0.25 µl of magnesium per specimen was utilized when amplifying the mitochondrial DNA (COI). The PCR mix underwent the time and temperature cycles listed, with different annealing temperatures for each targeted gene sequence as stated in Table 4. PCR products were cleaned and purified with either the QIAquick PCR Purification Kit (Qiagen, Inc.) or ExoSAP-IT (Affymetrix, Inc.). DNA concentrations were estimated by UV visualization of SYBR Safe (Invitrogen, Life Technologies) stained 1% agarose gel with Tris-borate-EDTA (TBE) electrophoresis buffer using standard techniques. Sequences were visualized and recorded using the Applied Biosystems (ABI) 3730xl Sequencer at the University of Minnesota Genomics Center. Each DNA fragment was sequenced from both directions. We also downloaded public COI sequences from the Barcode of Life Data Systems (BOLD) (Ratnasingham and Hebert 2007) for those taxa represented in our targeted enrichment data set.

**Table 3.** Primers used in polymerase chain reactions for this study.

Primer	Sequence (5’ to 3’)	Reference
COI F	TAATTGGAGGATTTGGWAAYTG	Kjer et al. 2001
COI R	CCYGGTAAAATTAAAATATAAACTTC	Kjer et al. 2001
D1 up	GGAGGAAAAGAAACTAACAAGGATT	Kjer et al. 2001
D1dn	CAACTTTCCCTTACGGTACT	Kjer et al. 2001
D2up4	GAGTTCAAGAGTACGTGAAACCG	Zhou et. al. 2007
D2dnB	CCTTGGTCCGTGTTTCAAGAC	Zhou et. al. 2007
D3up	ACCCGTCTTGAAACACGGAC	Kjer et al. 2001
D3DnTr2	CTATCCTGAGGGAAACTTCGGA	Kjer et al. 2001

**Table 4.** PCR settings (cycles, temperature, time) for each targeted gene sequence.

Repetitions	Temperature (°C)	Time
1 ×	94	3 minutes
40 ×	94	30 seconds
40 ×	52 – COI	30 seconds
40 ×	56 – D1	30 seconds
40 ×	57 – D2	30 seconds
40 ×	61 – D3	30 seconds
40 ×	72	30 seconds
40 ×	72	7 minutes
1 ×	4	hold

### ﻿Targeted enrichment analysis

Paired-end raw reads were delivered in FASTQ files by Rapid Genomics for the targeted enrichment taxa. We trimmed adapters from the raw reads using TrimGalore! (Babraham Bioinformatics 2019). We then followed the targeted enrichment analysis pipeline published by Breinholt et al. (2018). In brief, we assembled the trimmed reads into targeted gene sequences using iterative baited assembly. Then, for each gene targeted, we searched against the *Stenopsychetienmushanensis* reference genome assembly (Luo et al. 2018) with BLAST to assess orthology. If a selected gene generated multiple hits in the genome assembly, then that gene was removed from further analysis. We then assessed contamination in the data set by an all-by-all comparison with USEARCH v. 11 (Edgar 2010). If a hit was more than 98% identical over more than 80% of the gene sequence, both gene sequences were removed from further analysis. We combined orthologous sequences into unaligned FASTA files, which were aligned with MAFFT v. 7 (Katoh and Standley 2013) using the “AUTO” alignment setting.

### ﻿Alignment of Sanger sequencing data

Forward and reverse sequence fragments were edited and aligned in the program Geneious (Geneious Pro, v. 5.6.3, created by Biomatters). Consensus sequences for mitochondrial DNA (COI) were aligned using translation alignment in Geneious, while consensus sequences for ribosomal RNA (D1-3) were aligned using the MUSCLE alignment. Gaps and ambiguous sequences were coded as missing (-). Nucleotides were treated as unordered characters with four alternative states.

### ﻿Phylogenetic analysis

**Figure 2.**
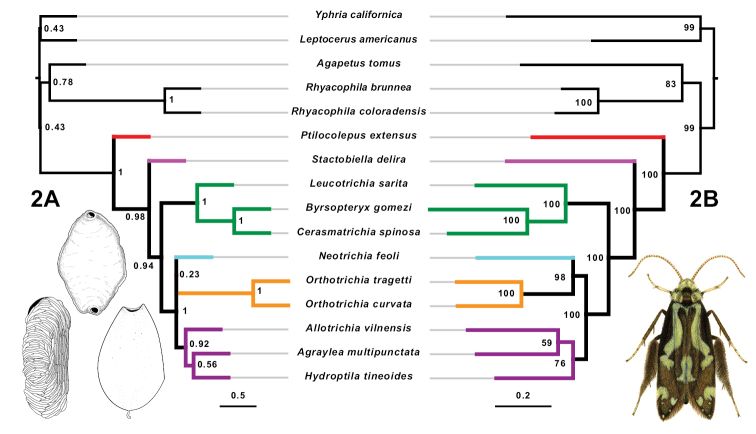
Targeted enrichment data only trees **A** astral multi-species coalescent tree. Support values are local posterior probabilities. Scale bar: coalescent units. Larval cases: *Leucotrichia* (top), *Dibusa* (left), *Ithytrichia* (right) **B** maximum-likelihood tree of concatenated supermatrix. Support values are ultra-fast bootstraps estimated in IQ-TREE. Scale bar: substitution rate. Adult: *Ascotrichia* sp.

**Figure 3.**
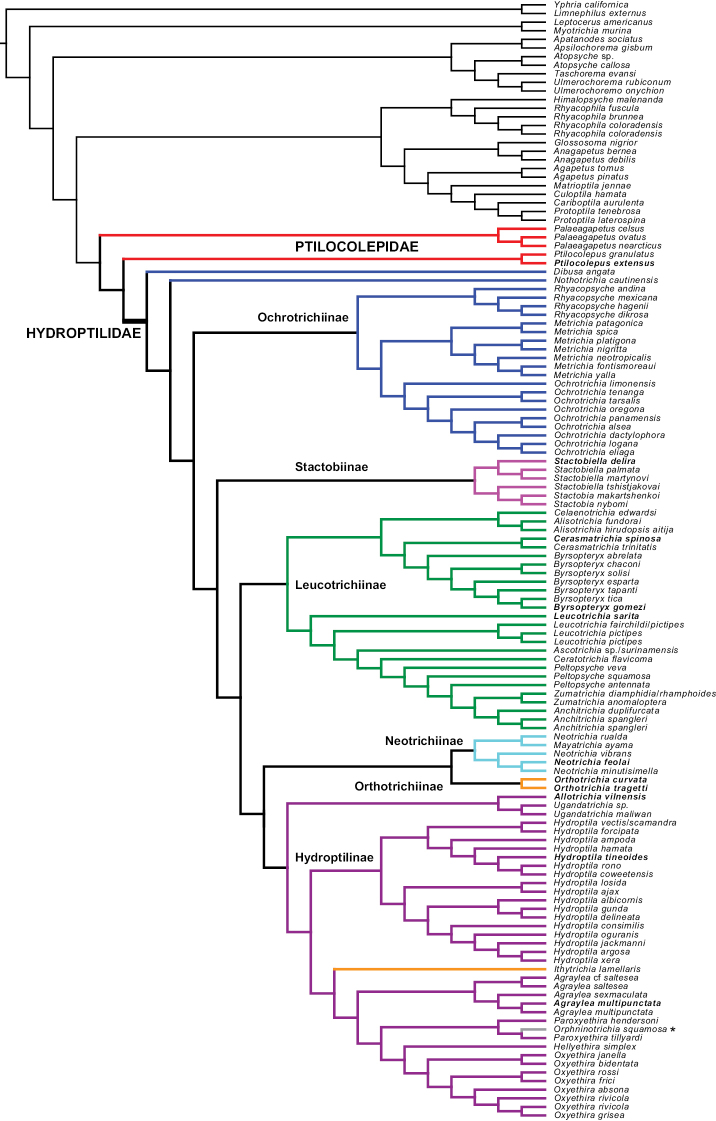
FastRFS majority-rule supertree derived from gene trees generated from both targeted enrichment data and Sanger sequencing data. Bold italic font indicates taxa that include targeted enrichment data. *Orphninotrichia, incertae sedis in current classification.

We generated three phylogenetic estimates from our data: (1) a maximum-likelihood tree based on a concatenated supermatrix of the targeted enrichment data (Fig. 2A), (2) a multispecies coalescent tree generated from maximum-likelihood trees of individual targeted enrichment loci (Fig. 2B), and (3) a fastRFS supertree based on the maximum-likelihood trees of individual targeted enrichment loci and the alignments from Sanger data of COI and 28S (Fig. 3). Single gene alignments and tree files were deposited in the Dryad Data Repository at https://doi.org/10.5061/dryad.15dv41p0n (Thomson et al. 2022).

Unfortunately, 100% of the gene fragments chosen for this study were not successfully sequenced for every species in the dataset. In a few situations, genera were represented by only a few species between which the recovered gene sequences did not overlap (ex: COI and D2 for Species 1, D1 and D3 for Species 2). In these instances, voucher material from the individual specimens was examined and identification was re-confirmed before combining the non-overlapping sequences as a single taxon, as indicated in Table 2.

To generate the maximum likelihood phylogenetic estimate for the supermatrix, we first concatenated the individual gene alignments into a concatenated supermatrix using FASconCAT (Kück and Meusemann 2010). We then used the FASconCAT info file to create an IQTREE partition definition file. We selected an optimal partitioning scheme using the relaxed clustering algorithm in IQ-TREE v.2.0.6 (Minh et al. 2020) with the options “-mset GTR -m TESTMERGEONLY”. We then selected the best fit substitution model for each subset in the partitioning scheme using ModelFinder as implemented into IQ-TREE v.2.0.6 with the option “-m MFP” (Minh et al. 2020). Using this model, we ran 25 separate maximum likelihood tree searches with 1000 ultrafast bootstrap replicates (option -bb 1000) and chose the tree with the best maximum-likelihood score (Hoang et al. 2017).

To generate a multi-species coalescent species tree, we first generated individual gene trees for each targeted enrichment locus with IQ-TREE v.2.0.6 (Minh et al. 2020). For each tree, we first selected the best substitution model with ModelFinder and then estimated 25 maximum likelihood trees with 1000 ultrafast bootstrap replicates and selected the tree with the maximum likelihood. We then used these trees as input for ASTRAL-III (Zhang et al. 2018).

Finally, we incorporated Sanger sequencing data for 28S and COI into a supertree analysis as described in Letsch et al. (2021). In their paper, they found that the supertree approach fastRFS (Vachaspati and Warnow 2017) generated the most reliable trees when combining Sanger sequencing data for many taxa with a “backbone” phylogenomic dataset that represented a smaller subset of those same taxa. Briefly, we concatenated the four PCR regions (D1, D2, D3 of 28S and COI) into a supermatrix and generated a tree using the same methods outlined above for the targeted enrichment loci. We then used fastRFS (Vachaspati and Warnow 2017) to estimate a “supertree” that considers both the targeted enrichment-based backbone tree and the increased taxon sampling made possible via the Sanger sequencing data.

## ﻿Results

### ﻿Summary of analyses

**Ptilocolepidae**

Only a single *Ptilocolepus* species was included in the targeted enrichment dataset, so no conclusions regarding the monophyly of Ptilocolepidae can be made based on the two targeted enrichment trees (Fig. 2A, B). In both targeted enrichment trees, however, *Ptilocolepus* was recovered as sister to Hydroptilidae (PP: 1, BS: 100).

*Palaeagapetus* and *Ptilocolepus* were each recovered as monophyletic in the fastRFS supertree (Fig. 3), although Ptilocolepidae was recovered as paraphyletic in relation to Hydroptilidae.

#### Hydroptilidae

A monophyletic Hydroptilidae was recovered in the target enrichment trees (PP: 0.98, BS: 100) and in the fastRFS supertree.

#### Hydroptilinae

In both targeted enrichment trees, Hydroptilinae formed a monophyly represented by one species each from the genera *Agraylea*, *Allotrichia*, and *Hydroptila* (PP: 0.92, BS: 76).

Hydroptilinae was not recovered as monophyletic due to the inclusion of species of *Ithytrichia* and *Orphninotrichia*. The genera *Hydroptila*, *Agraylea*, and *Oxyethira* were each recovered as monophyletic within Hydroptilinae, each represented by at least five species.

#### Leucotrichiinae

A monophyletic Leucotrichiinae was recovered in both targeted enrichment trees (PP: 1, BS: 100). The tribe Leucotrichiini was represented by only a single *Leucotrichia* species, so no conclusions regarding the monophyly of the tribe can be made. A monophyletic Alisotrichiini was also supported, based on a single species from each of the genera *Byrsopteryx* and *Cerasmatrichia* (PP: 1, BS: 100).

The fastRFS supertree also presented a monophyletic Leucotrichiinae and included a monophyletic Leucotrichiini sister to a monophyletic Alisotrichiini, with each tribe represented by at least four genera.

#### Neotrichiinae

Neotrichiinae was represented in the targeted enrichment dataset by only a single *Neotrichia* species, and thus no conclusions can be made on its monophyly. The single *Neotrichia* species appeared as sister to *Orthotrichia* in both trees, although with mixed support (PP: 0.23, BS: 98).

In the fastRFS supertree, Neotrichiinae was recovered as both monophyletic and sister to *Orthotrichia*. Neotrichiinae + *Orthotrichia* formed a clade sister to Hydroptilinae (if *Ithytrichia* and *Orphninotrichia* are included within Hydroptilinae).

#### Ochrotrichiinae

No targeted enrichment data representing members of the Ochrotrichiinae subfamily were available.

Based upon the genera currently included in Ochrotrichinae, the monophyly of the subfamily was not recovered in the fastRFS supertree. (*Metrichia* + *Ochrotrichia*) + *Rhyacopsyche* formed a distinct clade, but *Nothotrichia* and *Dibusa* failed to group with the rest of the ochrotrichiinae genera. Both latter two genera were recovered near the base of Hydroptilidae, with *Dibusa* sister to the rest of the hydroptilids.

#### Orthotrichiinae

Orthotrichiinae was represented by only a single genus, *Orthotrichia*, in the targeted enrichment dataset, and thus no conclusions regarding the monophyly of the subfamily can be made based on these trees. In both targeted enrichment trees, *Orthotrichia* formed a cluster with *Neotrichia* and Hydroptilinae (PP: 1, BS: 100).

The monophyly of Orthotrichiinae was not recovered in the fastRFS supertree. *Orthotrichia* was recovered as sister to Neotrichiinae, while *Ithytrichia* was represented by a single species and grouped within Hydroptilinae.

#### Stactobiinae

No conclusions regarding the monophyly of Stactobiinae can be made based on the total enrichment dataset, as only a single *Stactobiella* species was included. This *Stactobiella* was recovered as sister to the rest of Hydroptilidae (PP: 0.98, BS: 100).

A monophyletic Stactobiinae, represented by the genera *Stactobia* and *Stactobiella*, was recovered in the fastRFS supertree.

#### Incertae sedis

Of the genera currently considered incertae sedis within Hydroptilidae, only Sanger sequence data for a single species of *Orphninotrichia* was available.

In the fastRFS supertree, this *Orphninotrichia* species was grouped within the genus *Paroxyethira* within Hydroptilinae.

## ﻿Discussion

### ﻿Ptilocolepidae

The monophyly of Ptilocolepidae was not recovered in this study, but the 2 ptilocolepid genera did form a monophyletic unit with Hydroptilidae in the fastRFS supertree based on both targeted enrichment and Sanger sequencing data (Fig. 3), thus supporting a previously hypothesized Hydroptiloidea (Thomas et al. 2020). A monophyletic Ptilocolepidae was also not recovered in a previous study exploring the relationships among the families of Trichoptera (Holzenthal et al. 2007a). No members of Ptilocolepidae were represented in the recent Malm et al. (2013) study using molecular data to explore the relationships of the suborders within Trichoptera. Ptilocolepidae has thus far not been recovered as a monophyletic unit in any recent phylogenetic studies employing statistical analyses.

#### Hydroptilidae

The monophyly of Hydroptilidae was recovered in this study (Figs 2, 3).

#### Hydroptilinae

A monophyletic Hydroptilinae was recovered in this study in the targeted enrichment trees (Fig. 2A, B). Hydroptilinae was also recovered in the fastRFS supertree (Fig. 3), if the understanding of the subfamily is more loosely interpreted to potentially include the genera *Ithytrichia* and *Orphninotrichia*. It is possible that this represents the appropriate placement of these genera, as the current understanding of the placement of *Orphninotrichia* is uncertain, and Marshall did hypothesize that in the future *Ithytrichia* and *Orthotrichia* might no longer be considered a monophyletic Orthotrichiinae (Marshall 1979). Further sampling of both genera would help to make a more confident conclusion about their placement.

Hydroptilinae is a very diverse and widely distributed group, sequencing still more taxa would allow us to further resolve its topology. In her review, Marshall (1979) noted the group’s success in diversity and distribution and the very heterogeneous appearance of the subfamily when viewed as a whole. She also commented that the group could consist of three subgroups distinguishable by affinities in the male and female genitalia and the general appearance and habits of the larvae: the *Agraylea* group, the *Hydroptila* group, and the *Oxyethira* group. The potential for these three subgroups can be seen in the supertree, but additional sampling to include representation of more Hydroptilinae genera is needed.

#### Leucotrichiinae

The subfamily Leucotrichiinae was recovered in both the targeted enrichment trees and the fastRFS supertree. Additionally, the tribes Alisotrichiini and Leucotrichiini were also recovered as monophyletic sisters in the supertree, in agreement with Santos et al. (2016). This reinforces Marshall’s (1979) comment that, although the morphological boundaries of some of the leucotrichiine genera themselves are not always distinct and clear-cut, the subfamily itself does appear to form a unique clade within Hydroptilidae.

#### Neotrichiinae

The subfamily Neotrichiinae was recovered as monophyletic in the fastRFS supertree, but additional sampling to include more genera would help to strengthen this conclusion. In both the targeted enrichment trees and the supertree, Neotrichiinae, however represented, appeared as sister to *Orthotrichia*. Marshall (1979) included *Orthotrichia* as a member of Orthotrichiinae, but also mused that the genera included in that subfamily might be considered to be separate groups in the future. Additional sampling may help to resolve whether *Orthotrichia* truly is sister to Neotrichiinae, or should perhaps be considered as a member of the neotrichiine subfamily.

#### Ochrotrichiinae

Unfortunately, no targeted enrichment data were obtained for any member of Ochrotrichiinae. Within the fastRFS supertree, however, the genera *Metrichia*, *Ochrotrichia*, and *Rhyacopsyche* were recovered as a clade. When Ochrotrichiinae was first established by Marshall (1979), she stated that the features on which she based the group may one day prove to be secondarily derived from the general form of the Hydroptilinae and that Ochrotrichiinae may indeed prove to be a subgroup of Hydroptilinae. At least in this study, based on the three genera included in Marshall’s original Ochrotrichiinae, the evidence does not support this conjecture.

*Nothotrichia* and *Dibusa* did not form a monophyletic Ochrotrichiinae with the other three included genera. The genus *Nothotrichia* was originally left unplaced within Hydroptilidae by Marshall (1979); Harris and Armitage (1997) later added *Nothotrichia* to Ochrotrichiinae but stated that they were still attempting to determine synapomorphies for the group. Marshall also left *Dibusa* unplaced within Hydroptilidae, but noted similarities between *Dibusa*, *Nothotrichia*, and the hydroptiline genus *Agraylea* (1979); *Dibusa* was later added to Ochrotrichinae by Oláh and Johanson (2011), but no explanation for the inclusion was provided. Additional exploration is needed to determine if *Dibusa* and *Nothotrichia* should remain included in Ochrotrichiinae, or if they should be formally placed elsewhere.

#### Orthotrichiinae

The subfamily Orthotrichiinae was not recovered as a monophyletic unit. Nielsen (1948) considered the two genera for which Orthotrichiinae was originally established (*Ithytrichia* and *Orthotrichia*) to be derived from a common ancestor because of a large number of shared larval features. However, in Marshall’s (1979) opinion, while the larvae do share a number of morphological and behavioral similarities, both the larvae and adults are distinct for each genus and Orthotrichiinae might not be considered a cohesive unit. Additional sampling from both genera, and the potential inclusion of the third genus *Saranganotrichia*, may be necessary to understand the phylogenetic placement of Orthotrichiinae.

#### Stactobiinae

The subfamily Stactobiinae was recovered as monophyletic in the fastRFS supertree. Given previous researchers’ difficulty in finding morphological features that could be used to unite this group (Wells 1990; Bowles et al. 1999; Malicky and Chantaramongkol 2007), further work and detailed observations are needed to more clearly define this subfamily.

In the targeted enrichment trees, Stactobiinae was recovered as sister to the rest of Hydroptilidae, which was not in agreement with the arrangement of the fastRFS supertree. This discrepancy is likely due to the difference in taxon coverage between the targeted enrichment sequences and the Sanger sequences; additional targeted enrichment data sampled from across all six subfamilies may resolve this disagreement.

#### Incertae sedis

The genus *Orphninotrichia*, though only represented in this study by a single species, was recovered within a clade of hydroptiline genera (Fig. 3). This placement is independently corroborated by Marshall’s (1979) consideration that the genus shared similarities with other members of Hydroptilinae. There are two additional extant genera currently considered incertae sedis within Hydroptilidae, *Dicaminus* and *Macrostactobia*, but no sequence data was available for these. The three extinct incertae sedis genera, *Burminoptila*, *Electrotrichia*, and *Novajerseya*, cannot be placed using molecular data.

## ﻿Conclusions

The objectives of this paper were to provide a preliminary analysis 1) testing the monophyly of both Hydroptilidae and Ptilocolepidae, 2) evaluating the monophyly of the traditionally recognized subfamilies within Hydroptilidae, and 3) inferring relationships within and between Hydroptilidae, its included subfamilies, and Ptilocolepidae. This was the first study to explore a phylogenetic assessment of the family Hydroptilidae using modern statistical methods and molecular data. We show that an existing targeted enrichment probe set worked well on Hydroptilidae and provided strong support for the deeper relationships in the family. Further planned advancements of this study focusing on targeted enrichment data will confer taxonomic stability to the family, refine the current classification system, and provide a new phylogenetic framework in which to place new species and genera. Additionally, given the level of diversity and global distribution of Hydroptilidae, the extensive inclusion of more taxa may also produce a more strongly supported topology. A phylogenetic assessment of the relationships within the microcaddisflies will define the natural limits of the genera and subfamilies and their evolutionary relationships within the family, which in turn will support a stable classification of the hydroptilids. This provides an evolutionary framework in which to place undescribed microcaddisfly species, of which there are 100s, many of which occur in threatened ecosystems. It will also provide an evolutionary framework to investigate the unique life history features of the family, its diversity of larval case morphology, feeding strategies, male genitalia morphology, male secondary sexual characteristics, and patterns of regional endemism and other distributions.
